# Training health care workers to promote HIV services for patients with tuberculosis in the Democratic Republic of Congo

**DOI:** 10.1186/1478-4491-7-23

**Published:** 2009-03-17

**Authors:** Koen Vanden Driessche, Mulangu Sabue, Wendy Dufour, Frieda Behets, Annelies Van Rie

**Affiliations:** 1 School of Public Health, University of North Carolina, Chapel Hill, NC 27599-7435, USA; 2 Ecole de Santé Publique, Kinshasa, DR Congo

## Abstract

**Background:**

HIV counseling and testing, HIV prevention and provision of HIV care and support are essential activities to reduce the burden of HIV among patients with TB, and should be integrated into routine TB care.

**Methods:**

The development of training materials to promote HIV services for TB patients involved the definition of target health care workers (HCWs); identification of required tasks, skills and knowledge; review of international guidelines; and adaptation of existing training materials for voluntary counseling and testing, prevention of mother-to-child transmission of HIV, and management of opportunistic infections (OIs). Training effectiveness was assessed by means of questionnaires administered pre- and post-training, by correlating post-training results of HCWs with the centre's HIV testing acceptance rates, and through participatory observations at the time of on-site supervisory visits and monthly meetings.

**Results:**

Pre-training assessment identified gaps in basic knowledge of HIV epidemiology, the link between TB and HIV, interpretation of CD4 counts, prevention and management of OIs, and occupational post-exposure prophylaxis (PEP). Opinions on patients' rights and confidentiality varied. Mean test results increased from 72% pre-training to 87% post-training (p < 0.001). Important issues regarding HIV epidemiology and PEP remained poorly understood post-training. Mean post-training scores of clinic's HCWs were significantly correlated with the centre's HIV testing acceptance rates (p = 0.01). On-site supervisory visits and monthly meetings promoted staff motivation, participatory problem solving and continuing education. Training was also used as an opportunity to improve patient-centred care and HCWs' communication skills.

**Conclusion:**

Many HCWs did not possess the knowledge or skills necessary to integrate HIV activities into routine care for patients with TB. A participatory approach resulted in training materials that fulfilled local needs.

## Background

The World Health Organization (WHO) estimated that in 2005 alone there were approximately 8.8 million new tuberculosis (TB) cases and 1.6 million TB deaths, of which 195 000 occurred among people co-infected with the human immunodeficiency virus (HIV) [1]. The Democratic Republic of Congo (DRC) is ranked as the 11^th ^highest globally burdened by TB, with approximately 205 000 new cases annually, of which 20% are estimated to be among persons HIV co-infected [1,2].

In 2004, WHO published the interim policy on collaborative TB/HIV activities. Key activities are establishing mechanisms for collaboration, activities to decrease the burden of HIV in patients with TB and activities to decrease the burden of TB in people living with HIV/AIDS [3]. While many countries have developed training manuals for voluntary counseling and testing, prevention of mother-to-child transmission of HIV, antiretroviral treatment and treatment of opportunistic infections (OIs), no manuals for training in collaborative TB/HIV activities for health care workers (HCWs) at primary health care clinics could be identified in spring 2005 when scale-up of HIV services for TB patients was being planned for Kinshasa, capital of the DRC.

We aimed to develop and evaluate training materials for provider-initiated HIV counseling and testing, HIV prevention and integrated primary HIV care and support for HCWs involved in the care of patients with TB at the primary health care clinic level in the DRC.

## Methods

### Identification of target health care workers

Primary health care nurses play the central role in TB case management and were identified as the ideal HCWs to offer and provide HIV counseling and testing for TB patients and HIV care and support for those co-infected with HIV [2,4]. Other HCWs playing a key role in TB control, including laboratory technicians, physicians and district supervisors, were also identified as target HCWs. Furthermore, to avoid interruption of activities in case of illness, holidays or reassignments of HCWs, several other nurses from the primary health care centres were also selected for training.

HCWs participating in the training were employed at 14 primary health care clinics (under the direction of non-profit Protestant or Catholic organizations), selected among all 89 TB clinics in the capital based on criteria of annual TB case load, existence of some HIV activities and enthusiasm of the clinic director for project participation [5].

### Development of training materials

Training objectives and materials were developed in collaboration with educational specialists, DRC National HIV and TB Control Program officers, international TB experts and local HCWs. In a first step, we gained insight in tasks, skills and knowledge necessary for good performance from on-the-job experiences of nurses who provided HIV activities at TB clinics during pilot projects [2]. Second, we documented the DRC policy regarding HIV activities for patients with TB, which included provider-initiated HIV counseling and testing, HIV prevention, cotrimoxazole prophylaxis and referral for antiretroviral treatment (ART). Third, existing DRC training materials for HIV counseling and testing, prevention of maternal-to-child transmission of HIV and management of OIs were reviewed. Policy guidelines on collaborative TB/HIV activities and integrated management of adult illnesses were also consulted [3,6,7]. The new training materials were developed by focusing on topics that were highly relevant to tasks performed by primary HCWs. The training was also considered an opportunity to promote patient-centred care and to provide training on communication techniques for HCWs.

### Evaluating the newly developed training

Participants were asked to complete an assessment of their knowledge and attitudes pre-training and two weeks after the training. The structured questionnaire consisted of 38 multiple-choice questions concerning HIV transmission routes, natural history of HIV, epidemiology of HIV, the link between TB and HIV, interpretation of CD4 counts, HIV testing and counseling concepts, universal precautions, cotrimoxazole prophylactic treatment (CPT) and OI management, occupational post-exposure prophylaxis (PEP) and opinions on patients' rights and confidentiality. To gain further insights, participants were also asked to provide an explanation for their answer to several key questions.

Questions addressing the same topic were grouped to calculate a mean test result for each topic. A topic was believed to be sufficiently understood when the proportion of correct answers was at least 80%. Pearson's Chi-square test allowed comparison of proportions of correct answers pre- and post training. Post-training scores of clinic's HCWs were compared with the clinic's HIV testing acceptance rate by calculating the Pearson correlation coefficient.

An educational expert and staff from the national HIV and TB programmes (n = 5) together with an expert in collaborative TB/HIV care, one experienced laboratory technician and six HIV counsellors participated in the training sessions to provide feedback. Revision of the training materials was based on results of the observations made during the training and the results of the pre-and post-training assessments of the participants' knowledge and attitudes.

Participatory observations during on-site supervisory visits and monthly meetings were planned as an integral part of the training. These activities helped to ensure that tasks were performed as intended by the training. Supervision was an opportunity to correct errors because of incomprehension or unforeseen circumstances, and to morally support and motivate the HCW. Supervision was done by a laboratory technician and four HIV counselors who participated in the development and assessment of the training. A treatment card for collaborative TB and HIV activities was introduced to record TB/HIV activities and helped to identify weakness in the performance that needed attention. The study was reviewed by the Institutional Review Board of the University of North Carolina.

## Results

### Pre-training knowledge and opinions among HCWs on TB and HIV

**Figure 1:**
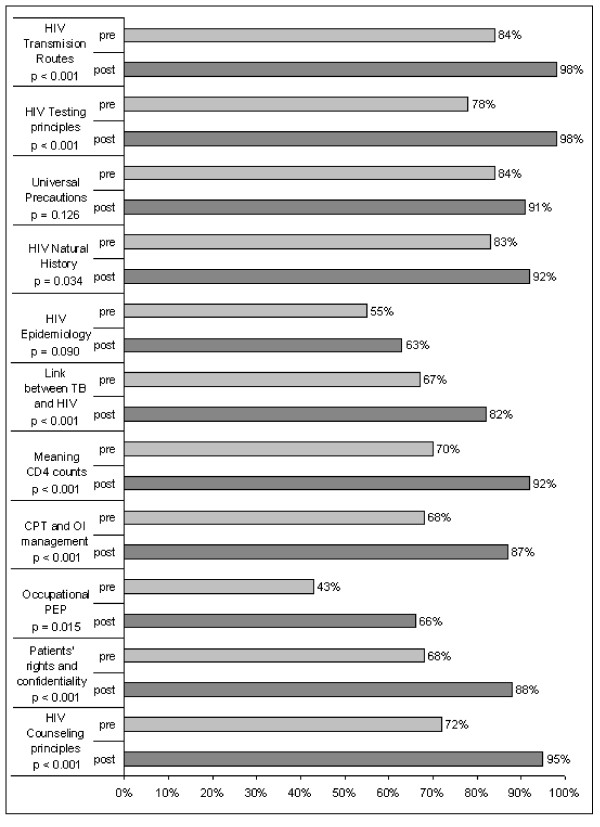
**Pre- and post-training HIV and TB knowledge among 67 health care workers involved in TB management in Kinshasa, DRC**. p-values were obtained by Chi-square testing for difference in proportions. HIV: human Immunodeficiency virus; TB: tuberculosis; CD4: CD4-positive lymphocytes; CPT: co-trimoxazole prophylactic treatment; OI: opportunistic Infection; PEP: post-exposure prophylaxis

Two groups of HCWs (total n = 67) completed the pre-training assessment (Fig 1). While most HCWs had adequate knowledge of HIV transmission routes, 7% of participants believed HIV was not transmitted through breast milk and 3% answered that HIV could be transmitted via mosquito bites. Questions on important concepts such as the window period in HIV testing, universal precautions and the natural history of HIV were answered correctly by most HCWs.

Important gaps were identified and included knowledge of HIV epidemiology, the link between TB and HIV, the meaning of CD4 counts, principles of CPT, OI management and occupational PEP (Fig 1). The majority (56%) of participating HCWs substantially underestimated the burden of HIV in Africa, with 36% participants being convinced that only 10% of people living with HIV reside in Africa. In contrast, the HIV prevalence in DRC was overestimated by almost half (45%) of HCWs, with 15% answering that more than 30% of the DRC population was infected with HIV, which is almost 10-fold the Joint United Nations Programme on HIV/AIDS (UNAIDS) HIV prevalence estimate of 3.2% for 2005 [8]. Only 11 (16%) participants answered correctly that, in the DRC, HIV prevalence is higher among TB patients than in the general population. Less than half (43%) of the HCWs knew that lower CD4 counts are associated with more severe immunosuppression. Fourteen (21%) participants did not know that CPT should be continued when anti-tuberculosis treatment is completed. Only 29 (43%) chose to first clean the lesion in case of a needle-stick injury.

Opinions on patients' rights and confidentiality varied. Most HCWs (48, or 72%) believed that a patient's HIV test result could not be divulged to a colleague when referring the patient for care (OI treatment), because the test result is a medical secret. When asked if the HCW has to test all TB patients for HIV, 44 participants (66%) answered yes, 21 participants (31%) answered no and two participants left the question open. Most believed this was: "to ensure good care and follow-up of our patients even after the treatment of tuberculosis", but two participants answered "because the HIV test is obligatory" and three others answered "to develop statistics on HIV among TB patients". Fifteen (22%) participants did not agree that a HCW can accept a test refusal because: "Refusing an HIV test is equivalent to keeping the patient ignorant and that is discrimination" and "It is for the patient's own good. The patient should know his serology so he can better protect himself and prolong his stay on earth."

### Content of the training modules

Table 1 details the content of the training. Topics included HIV epidemiology, transmission modes and natural course of HIV infection, HIV prevention within the health care setting (universal precautions), the link between TB and HIV, the WHO policy on collaborative TB/HIV activities, provider-initiated HIV counseling and testing in the TB clinic, care for HIV co-infected TB patients, and monitoring and evaluation of HIV activities, including the use of a modified TB treatment card. Training on the management of HIV co-infected TB patients focused on CPT, nutritional education and psychosocial support. Training on management of OIs focused on care feasible at primary health care level and indications for referral, rather than extensive training on diagnostics. Modules on ART were not included because of the policy to refer patients for ART and the extremely limited access to and experience with ART for patients with TB in the DRC [1].

**Table 1:** Content of training modules for TB/HIV collaborative activities at the primary health care level.

**FIRST TRAINING DAY**
Introduction

Module 1: HIV infection

- Differences between HIV and AIDS

- The epidemiology of HIV in the world and DRC

- The immune system and the natural course of HIV infection

- HIV transmission modes

Module 2: Preventing HIV infection

- HIV prevention methods

- Prevention of HIV within the health care setting

Module 3: TB/HIV co-epidemic

- The epidemiology of TB

- The link between TB and HIV

- Reducing the burden of HIV among TB patients

**SECOND TRAINING DAY**

Module 4: HIV diagnosis

Module 5: HIV counseling and testing in TB treatment centers

- Four types of HIV counseling and testing

- Provider-initiated diagnostic counseling and testing

- Key responsibilities and attitudes of the TB/HIV counselor

- The importance of the 3 Cs in HIV testing

- Basic counseling techniques

- Practice by role play

Module 6: Pre-test counseling for TB patients

- Stages of diagnostic pre test counseling

- Practice by role play

**THIRD TRAINING DAY**

Module 7: Post-test counseling for TB patients and psychosocial support

- Stages of post-test counseling according to HIV test result

- Practice by role play

- Psychosocial support

Module 8: Patient flow, recording, monitoring and evaluation

**FOURTH TRAINING DAY**

Module 9: Management of HIV co-infected TB patients

- Role of the TB service: opportunities and challenges

- Co-trimoxazole preventive therapy

- Nutritional education

- Management of opportunistic infections and other medical problems linked to HIV (Respiratory problems – Digestive problems/diarrhoea – Neuropsychiatric manifestations – Dermatological manifestations – Oral lesions – Lymphadenopathies – Fever)

- Referral for care and support

- Introduction to ARV treatment

**MONTHLY MEETINGS**

Peer discussion and problem solving

Introduction of a specific topic

Module 10: Couple and family counseling

Module 11: HIV counseling of infants, children and adolescents

Module 12: Stigmatization and discrimination

Module 13: Support groups

Module 14: Community mobilization

Module 15: Palliative care

Topics were introduced using PowerPoint^® ^presentations, interactive question-and-answer sessions, group discussions and case studies, either with the entire group or in small breakout sessions. HIV counseling was demonstrated by health care workers experienced in these activities, followed by practice during role-play sessions in small groups such that trainees could actively acquire the new skills. Trainers gave immediate feedback on trainees' performance during these sessions.

The training materials in French, consisting of a participant's manual, a trainer's manual, Power Point^® ^slides, a training evaluation questionnaire and the revised treatment card can be obtained from the corresponding author.

### Experience with continuing education and participatory problem solving

Continuing education, motivation and problem solving occurred during on-site supervisory visits and monthly meetings with HCWs actively involved in implementing HIV activities for patients with TB. Project staff noticed that, even though the training included role playing to familiarize HCWs with pre- and post test HIV counseling, several HCWs felt uncertain about their skills. This problem was resolved by the presence of trainers on-site during the first "real life" HIV counseling session. HCWs also struggled with the reorganization of their daily work schedule, a necessary step for efficient integration of HIV counseling and testing into routine patient care. This was resolved through on-site discussions with the HCW, and re-evaluated on the next supervisory visit.

Monthly meetings were an opportunity for continuing education, peer discussion and problem solving. Training modules for these meetings focused on specific issues such as family counseling, approach to counseling of minors, causes and effects of stigmatization, role of support groups for HIV co-infected patients, community participation in HIV and TB prevention and palliative care. Discussions among HCWs at the meetings mostly concerned approaches to patients who refuse HIV testing at TB diagnosis, counseling strategies for patients who refuse to accept their HIV status, management of patients who default CPT and strategies to help patients disclose their HIV status to family members.

### Evaluation of the training and revision of the training manual

**Figure 2:**
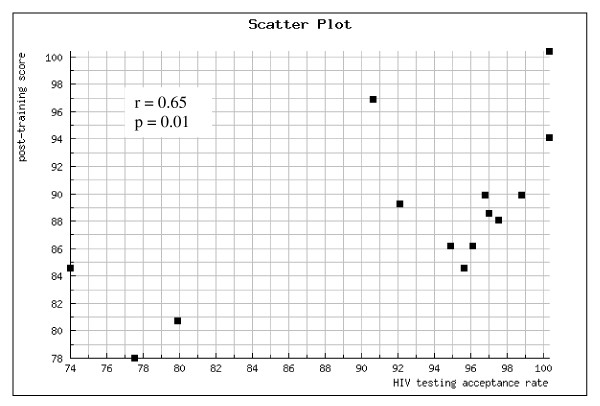
**Correlation between mean post-training scores and HIV testing acceptance rate (first 3 months of implementation of HIV activities for TB patients) at 14 primary health care clinics in Kinshasa, DRC**.

High training participation rates were achieved (91% to 100%) at all four consecutive Saturday training sessions and the training received positive feedback from participants. Sixty-five (97%) participants completed the post-training assessment, including 38 nurses, 16 laboratory technicians, 7 physicians and 4 district supervisors. The mean test score increased from 72% pre-training to 87% post-training (p < 0.001). There was no statistically significant difference in post-training score by type of HCW (p = 0.19), with a mean post-training score of 87% for nurses, 86% for laboratory technicians, 89% for physicians and 92% for district supervisors. The mean post-training scores of clinic's HCWs were significantly correlated with the clinic's HIV testing acceptance rate (Fig. 2).

Post-training, HCWs demonstrated significantly increased and adequate knowledge of HIV transmission routes, HIV counseling and testing principles, natural history of HIV, link between TB and HIV, meaning of CD4 counts, CPT and management of OIs, patients' rights and the importance of confidentiality (Fig 1). The only topics for which the proportion of correct answers remained below 80% were related to HIV epidemiology and PEP. One third of participants continued to have difficulties in estimating the burden of HIV and TB/HIV co-infection in the DRC and sub-Saharan Africa. Only 43 (66%) participants answered the PEP questions correctly.

Results of pre- and post-training assessments and observations made during the training guided the revision of the initial training manual. Topics that raised confusion or remained insufficiently understood were revised and more time was allocated to these topics in the revised modules. The revised modules were approved by the DRC National TB Program and used in roll-out of provider initiated HIV counseling and testing for TB patients in the DRC.

## Discussion

The HIV and TB co-epidemic is one of the most important public health problems in the African region. Activities to reduce the burden of HIV among TB patients and activities to reduce the burden of TB among people living with HIV/AIDS are therefore urgently needed [3]. HIV counseling and testing of patients with TB and care for co-infected patients are key activities in the fight against HIV and TB. We observed that in Kinshasa, DRC, the vast majority of HCWs actively involved in TB care did not possess sufficient knowledge or skills to integrate HIV services in TB clinics, confirming the need for specific training materials. We developed training materials with careful consideration of the primary health care clinic HCWs' tasks, organized the necessary knowledge acquisition around these tasks and used methods that actively involve trainees. The newly developed training materials filled the identified gaps in knowledge but came short in transferring counseling skills. However, combined with logistical support, on-site supervisory visits and monthly continuing education meetings, the training allowed the scale-up of HIV activities for patients with TB at primary health care clinics.

The correlation between mean post-training scores of clinic's HCWs and the clinic's HIV testing acceptance rate suggests that the training was effective. But providing a training course is only one of the many factors needed to achieve high HIV testing acceptance rates. Other factors that may play a role include logistic factors, HCW motivation and the levels of TB and HIV stigma in the community [9].

To reinforce the HCWs' capacity in patient management beyond specific HIV and TB services, we used the training as an opportunity to integrate concepts of patient-centred care and communication skills. Communication between patients and primary care providers is often poor in clinical practice in developing countries [10]. In rural DRC, health centre clients identified good interpersonal skills as the most important quality for nurses, followed by good competence and technical skills [11]. Promoting effective interpersonal communications during training thus has the potential to ensure both a high quality of services and their use by health centre clients [11].

Some limitations to the study should be noted. First, participating HCWs were employed at selected primary health care clinics in the capital. It is possible that the knowledge prior to the training was higher and not representative of all HCWs involved in TB care in Kinshasa. Furthermore, HCWs in the capital are more likely to have been exposed to HIV and TB messages and training compared to nurses employed in more rural areas. Knowledge of HCWs in the capital is thus most likely not representative of the entire country. Second, the post-training assessment evaluated participants' knowledge two weeks after the training, which is no guarantee of long-term knowledge retention. Finally, while we assessed the individual's acquisition of knowledge and the clinic level implementation of the new activities, no formal assessment of an individual's acquisition of skills was performed at the end of the training and the quality of task performance was also not formally assessed during on-site visits [12]. Several HCWs needed additional one-on-one training during the first "real life" HIV counseling session, which argues for further increasing the focus on transfer of skills, especially counseling skills.

## Conclusion

Integration of HIV activities into routine TB patient care is urgently needed, but HCWs often do not possess the knowledge and skills necessary to implement these activities. The newly developed training was effective in transferring this knowledge, and was also used as an opportunity to transfer concepts of patient-centred care and communication skills in order to improve patient management beyond HIV and TB care in this resource-poor setting. Involvement of the National TB and HIV Program staff in the development phase facilitated the use of the training materials by the National Program in the roll-out of TB/HIV training for HCWs involved in TB care in the DRC soon after completion of the final modules.

## Abbreviations

ART: Antiretroviral treatment; CPT: Cotrimoxazole prophylactic treatment; DRC: Democratic Republic of Congo; HCW: Health care worker; HIV: Human immunodeficiency virus; OI: Opportunistic infection; PEP: Post-exposure prophylaxis; TB: Tuberculosis; UNAIDS: Joint United Nations Programme on HIV/AIDS; WHO: World Health Organization.

## Competing interests

The authors declare that they have no competing interests.

## Authors' contributions

KVD developed the training, collected and analysed the data and drafted the manuscript. MS carried out the training for the TB health care workers and helped with its development. WD and FB also helped with developing the training. AVR conceived of the study, participated in developing the training, coordinated the study with FB and helped to draft the manuscript. All authors read and approved the final manuscript.
